# Multi-Frame Temporal Integration for 3-D Shape Measurement of Freely Falling Small Objects Using a High-Speed Camera Array

**DOI:** 10.3390/s26113457

**Published:** 2026-05-30

**Authors:** Hao Duan, Shaopeng Hu, Feiyue Wang, Kohei Shimasaki, Idaku Ishii

**Affiliations:** 1 Graduate School of Innovation and Practice for Smart Society, Hiroshima University, Higashi-Hiroshima Campus, Higashihiroshima 739-0046, Japan; hao-duan7650@hiroshima-u.ac.jp; 2 Digital Manufacturing Education and Research Center, Hiroshima University, Higashi-Hiroshima Campus, Higashihiroshima 739-0046, Japan; hsp@hiroshima-u.ac.jp; 3 Graduate School of Advanced Science and Engineering, Hiroshima University, Higashi-Hiroshima Campus, Higashihiroshima 739-0046, Japan; feiyue@hiroshima-u.ac.jp (F.W.); simasaki@hiroshima-u.ac.jp (K.S.)

**Keywords:** high-speed imaging, multi-frame reconstruction, volumetric sensing, millimeter-scale 3-D shape measurement

## Abstract

Dynamic three-dimensional (3-D) reconstruction of small objects moving at high speed is fundamentally limited by the number of viewpoints that a fixed camera array can provide at any single time instant. When the camera count is insufficient, single-frame multi-view stereo produces incomplete or inaccurate geometry. This paper proposes a multi-frame temporal integration approach that overcomes this limitation by exploiting the rigid-body assumption: because a falling object maintains its shape across consecutive frames, images captured at different time instants can be combined into a single, viewpoint-enriched reconstruction. A three-layer circular array of 32 synchronized RGB cameras captures 1440 × 1080 images at 160 fps, and a free-fall-oriented algorithm automatically detects active frames, selects informative temporal windows, and feeds the accumulated multi-frame images into a structure-from-motion and multi-view stereo (SfM-MVS) pipeline, effectively multiplying the number of viewpoints without additional hardware. The algorithm simultaneously recovers the 6-DOF pose trajectory of each object from the SfM-estimated camera parameters. Progressive accumulation experiments on freely falling soybeans (approximately 9–10 mm diameter) show that a single 32-camera frame already achieves an F-score exceeding 0.97 at a 0.5 mm threshold against an industrial structured-light scanner reference, and that accumulating additional temporal frames reaches a stable convergence plateau with both objects reaching a plateau F-score of 0.984. Beyond approximately one to two accumulated frames, additional frames yield diminishing returns, confirming that a small number of temporal frames is sufficient for convergent sub-millimeter accuracy. Across 30 independent free-fall trials with three objects, the system achieves an overall mean error of 0.146±0.033 mm and an overall F-score of 0.980±0.006—a mean relative error of approximately 1.6% on 8–10 mm targets—and fine surface features such as structural cracks are resolved at a fidelity sufficient for visual defect identification. These results establish rigid-body multi-frame temporal integration as an effective strategy for high-throughput, non-contact 3-D inspection of small objects in motion.

## 1. Introduction

Three-dimensional (3-D) reconstruction from multi-view images is a well-established technique whose accuracy depends critically on the number, distribution, and geometric diversity of the input viewpoints. The foundational benchmark by Seitz et al. [1] demonstrated a clear relationship between viewpoint count and reconstruction completeness, showing that increasing the number of views from 16 to 317 progressively improves surface coverage and geometric detail. This dependence is well understood theoretically: the visual hull [2] converges to the true shape only as the number of silhouette viewpoints approaches infinity, and practical multi-view stereo (MVS) algorithms require sufficient baseline diversity to resolve depth ambiguities [3,4]. For static objects, viewpoint sufficiency can be addressed by capturing many images from planned positions [5], but for dynamic scenes—where objects move continuously and each time instant provides only a single snapshot from a fixed camera array—the number of available viewpoints is physically constrained by the camera count.

In dynamic scenes, the viewpoint count of a fixed camera array sets an upper bound on reconstruction quality at any single time instant. Kanade’s Virtualized Reality dome [6] and subsequent performance capture systems [7,8,9] demonstrated that synchronized multi-camera arrays can capture dynamic events, but these systems typically employ dozens to hundreds of cameras to maximize viewpoint density. In compact or cost-sensitive configurations where the camera count is limited, a single frame can already produce a usable reconstruction, yet the achievable surface coverage and geometric detail remain bounded by the available viewpoint diversity. Simply adding more cameras is often impractical due to physical space constraints, synchronization complexity, and cost; an alternative strategy that increases effective viewpoint density without additional hardware is therefore desirable.

Several strategies have been explored to mitigate viewpoint insufficiency without increasing camera count. Learning-based MVS methods such as MVSNet [10] and its successive extensions—including recurrent depth inference [11], cascade cost volumes [12], learned patchmatch propagation [13], transformer-based global context aggregation [14], unified depth representations [15], and iterative probability estimation [16]—use data-driven depth priors to improve reconstruction from sparse views. Neural rendering approaches—including NeRF [17] and its extensions to unbounded scenes [18] and accelerated training [19], neural implicit surface methods [20,21], and high-fidelity neural surface reconstruction [22]—can synthesize novel viewpoints or recover geometry from limited input. Three-dimensional Gaussian Splatting (3DGS) [23] and its geometry-focused variants [24,25] enable real-time rendering with explicit point-based representations, and recent feed-forward variants such as pixelSplat [26] and MVSplat [27] can predict 3-D Gaussians from as few as two images in a single forward pass, while InstantSplat [28] eliminates the need for structure from motion by jointly optimizing Gaussians and camera poses from sparse views. More recently, Gao et al. [29] proposed a lightweight 16-camera system for free-fall multi-view reconstruction of coarse aggregate particles using implicit neural representation, with ray marching and multi-resolution hash encoding, reporting an average measurement error of 75 μm at a throughput of up to four particles per second. Their work shares the free-fall multi-view imaging premise, but uses an implicit neural reconstruction paradigm rather than a classical SfM–MVS pipeline, and does not exploit temporal redundancy under the rigid-body assumption. However, all these methods require either per-scene optimization or large-scale training data, and their applicability to high-speed industrial inspection of numerous small objects remains undemonstrated. For non-rigid scenes, temporal fusion methods such as DynamicFusion [30] and Fusion4D [31] accumulate depth information across frames using deformable tracking, and de Aguiar et al. [32] demonstrated that sparse multi-view video, combined with temporal coherence constraints can reconstruct human performance. More recently, neural deformable representations such as Nerfies [33] and dynamic Gaussian approaches [34,35] have extended temporal fusion to photorealistic quality. However, all these deformable approaches require complex non-rigid tracking and are designed for subjects whose shape changes continuously over time.

Beyond algorithmic approaches, advances in camera miniaturization and computational performance have enabled dense multi-camera arrays for volumetric capture across multiple spatial scales. At the microscopic level, selective plane illumination microscopy [36] and light-field microscopy [37] provide volumetric imaging of biological specimens, while neural rendering has recently been combined with atomic force microscopy for submicron 3-D reconstruction [38]. At the mesoscopic level, multi-camera array systems such as MCAM demonstrated gigapixel-scale imaging with synchronized cameras for wide-field volumetric tracking [39,40]. Building on this foundation, 3D-RAPID [41] and RUSH3D [42] achieved real-time gigapixel reconstructions at over 100 volumes per second, while MCAS enabled rapid 3-D digitization of pathology slides [43], and compact configurations such as the M25 multifocus microscope [44] and ReFLeCT [45] provided high-speed 4-D reconstructions of small organisms. These systems demonstrate the power of multi-camera arrays for biological and biomedical imaging, but their designs prioritize field-of-view coverage and fluorescence detection over the requirements of industrial inspection, where continuous object flow, complete 360° coverage, and high-throughput reconstruction of numerous rigid objects are essential.

A fundamentally simpler situation arises when the target object is rigid. Under the rigid-body assumption, images captured at different time instants depict the same invariant geometry observed from different viewpoints, because the object’s motion relative to the cameras changes the effective viewing angles. This principle has deep roots in computer vision: Tomasi and Kanade’s factorization method [46] showed that rigid shape and motion can be jointly recovered from tracked features across multiple frames, effectively treating temporal frames as additional viewpoints of a static structure. Belongie and Wills [47] exploited temporal periodicity to reconstruct rigid objects from monocular video, where period-separated frames serve as surrogates for multi-view observations. More recently, Abuzaina et al. [48] demonstrated motion-compensated temporal accumulation for 3-D reconstruction of rotating rigid objects from depth sensors. These works establish that, for rigid targets, temporal frames can directly augment the spatial viewpoint set—a principle that has not yet been applied to high-speed multi-camera arrays for industrial inspection of small objects.

Industrial inspection of small objects, such as seeds, electronic components, and mechanical parts, demands high-throughput, non-contact 3-D measurement with complete 360° coverage and sufficient accuracy to detect sub-millimeter surface defects. Conventional structured-light scanners, such as the ATOS Compact Scan (Carl Zeiss GOM Metrology) [49], achieve micrometer-level accuracy but require physical fixturing, background markers, and manual post-processing, making them fundamentally incompatible with continuous in-line inspection. Free-fall-based capture eliminates all contact constraints, but introduces the challenge of reconstructing fast-moving objects from a limited number of cameras within millisecond exposure windows.

To address this challenge, this study proposes a multi-frame temporal integration algorithm for 3-D shape measurement of freely falling small objects using a high-speed camera array. A three-layer circular array of 32 synchronized RGB cameras operating at 1440 × 1080 resolution and 160 fps captures omnidirectional images of objects in free fall. The proposed algorithm exploits the rigid-body assumption to accumulate images across multiple consecutive frames into a single SfM-MVS reconstruction, effectively multiplying the number of viewpoints from 32 (single frame) to 96 or more (three or more accumulated frames) without additional hardware. The algorithm further recovers the 6-DOF pose trajectory of each object from the SfM-estimated camera parameters.

The remainder of this paper is organized as follows. Section 2 details the hardware configuration of the three-layer camera array. Section 3 describes the multi-frame reconstruction algorithm, including active-frame detection, temporal accumulation under the rigid-body assumption, and 6-DOF pose estimation. Section 4 presents experimental validation using freely falling soybeans, quantifying the relationship between accumulated frame count and reconstruction accuracy, evaluating shape fidelity against an industrial reference scanner, and demonstrating surface feature preservation and full kinematic analysis.

## 2. High-Speed Camera Array System for Falling Small Objects

**Figure 1:**
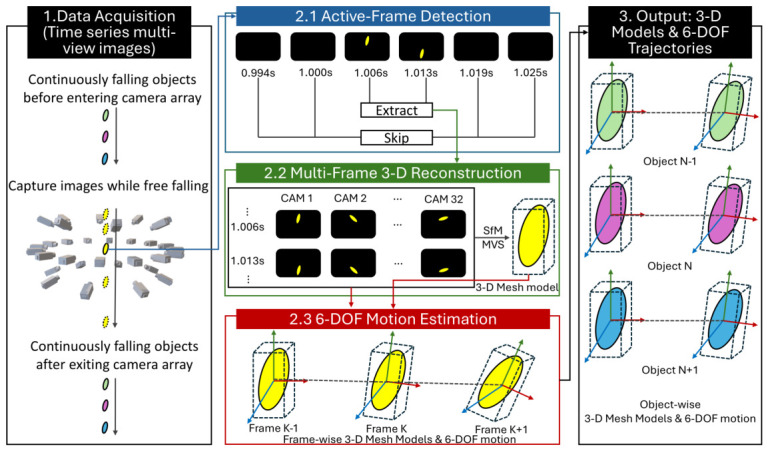
Conceptual overview of the proposed system for high-speed 3-D measurement of freely falling small objects. The pipeline consists of three stages: (1) data acquisition via a 32-camera synchronized array capturing multi-view image sequences at 160 fps; (2) algorithmic processing comprising active-frame detection, multi-frame 3-D reconstruction via SfM–MVS, and 6-DOF motion estimation; and (3) dual outputs of object-wise 3-D mesh models and frame-wise 6-DOF trajectories.

Figure 1 presents a conceptual overview of the proposed system. The core design principle is that a freely falling object traverses the camera array within milliseconds, during which 32 cameras simultaneously capture it from all azimuthal directions. By accumulating images across multiple consecutive frames, the effective viewpoint coverage is substantially increased beyond the physical camera count, enabling high-fidelity 3-D reconstruction and 6-DOF pose estimation from a single free-fall event. The following subsections detail the hardware components, while Section 3 describes the reconstruction algorithm.

**Figure 2:**
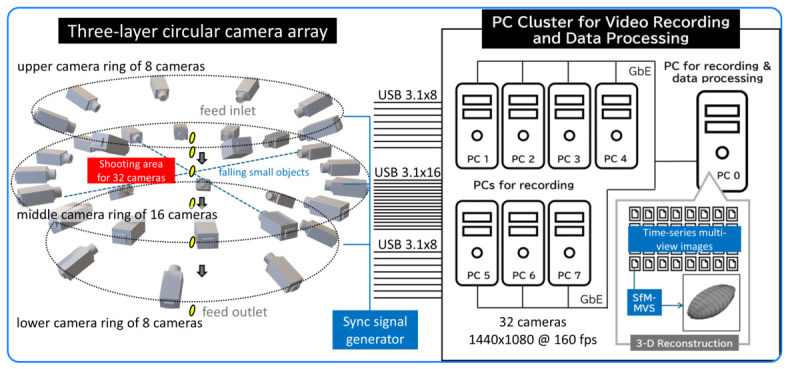
Hardware configuration of the high-speed camera array system. The three-layer circular camera array surrounds the central free-fall path, and a PC cluster manages synchronized video acquisition and offline 3-D reconstruction.

In order to achieve high-throughput industrial inspection of small objects, a volumetric imaging system must be capable of accommodating continuous feeding while ensuring occlusion-free capture. Conventional volumetric capture systems, however, rarely consider the feeding process, such as the inlet and outlet through which numerous objects must pass, and typically neglect the simultaneous observation of both top and bottom views. To address these requirements, this study employs a circular camera array composed of 32 high-speed USB cameras arranged in three layers around the free-fall path of small objects, enabling comprehensive 3-D reconstruction with complete coverage [50]. The system is supported by a PC cluster for high-frame-rate video recording and data processing. The overall configuration is illustrated in Figure 2.

**Figure 3:**
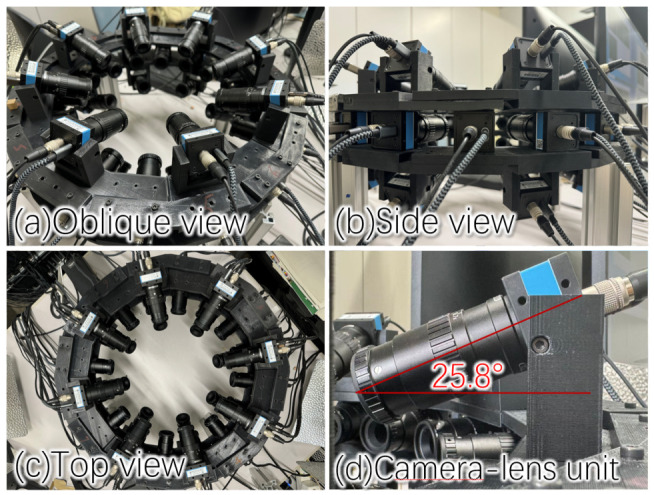
Overviews of the three-layer circular camera array: (**a**) oblique view of all 32 cameras; (**b**) side view showing the three layers; (**c**) top view showing the three layers; and (**d**) close-up of a single camera on an adjustable bracket.

The three-layer circular camera array, illustrated in Figure 3, consists of 32 USB 3.1 RGB cameras (DFK 37BUX273, Imaging Source GmbH, Bremen, Germany) arranged in three circular layers, each equipped with a 1440 × 1080-pixel CMOS color sensor with a pixel pitch of 3.45 × 3.45 μm. The cameras are distributed across two concentric circles on the base, with an inner circle of 315 mm diameter and an outer circle of 435 mm diameter: the upper and lower rings each contain 8 cameras uniformly spaced at $45^{\circ }$ intervals, while the middle ring contains 16 cameras uniformly spaced at $22.5^{\circ }$ intervals. Adjustable mounting brackets provide two degrees of rotational freedom, allowing precise alignment of each camera. Adjacent layers are vertically separated by 68 mm, forming a stereoscopic three-layer configuration. Each camera is equipped with a C-mount macro lens (ML-MC35HR, Moritex Corp., Kanagawa, Japan) and tilted such that the optical axes converge toward the geometric center of the array. At a distance of approximately 22 cm from the cameras near the ring center, a 1440 × 1080-pixel image corresponds to an area of about 18 × 13 mm, yielding a spatial resolution of approximately 13 μm/pixel. The falling objects are fed from above and move downward along a straight free-fall path that passes through the common center of the three camera rings. All 32 cameras are simultaneously triggered to capture the objects at a common observation point located at the geometric center of the three-layer array, with the upper and lower ring cameras tilted by 25.8° and the middle ring cameras kept essentially horizontal (∼$0^{\circ }$ tilt), so that all optical axes converge at this point. This configuration defines a common measurement volume of approximately 10 mm on each side, located along the central free-fall path of the objects through the camera array, where they are captured from all directions with minimal occlusion.

The PC cluster consists of eight computers: PC0 serves as both the recording and processing node, while PC1–PC7 are dedicated to video recording. Their hardware configurations are listed in Table 1. All cameras are triggered simultaneously by an external function generator (AFG1062, Tektronix, Beaverton, OR, USA), and images are recorded locally at 160 fps in uncompressed BMP format. The maximum continuous recording duration is approximately 7 s, limited by the 32 GB RAM of PC3–PC7. After recording, all image data are transferred via Gigabit Ethernet to PC0, which performs offline SfM–MVS reconstruction using its high-end GPU.

## 3. Multi-Frame 3-D Capture Algorithm for One-by-One Falling Objects

As established in Section 2, a fixed 32-camera array provides 32 viewpoints per time instant, and while this is sufficient for a usable reconstruction, increasing the effective viewpoint density can further improve surface coverage and geometric detail. The key insight of the proposed algorithm is that freely falling objects can be treated as rigid bodies, so that images captured at different time instants depict the same invariant geometry from different effective viewpoints. By accumulating images across multiple consecutive frames, the number of input viewpoints is multiplied beyond the physical camera count without additional hardware. Temporal sparsity is further exploited: blank frames constitute the majority of captured images, and only active frames containing the object are selected, enabling efficient SfM-MVS processing.

**Figure 4:**
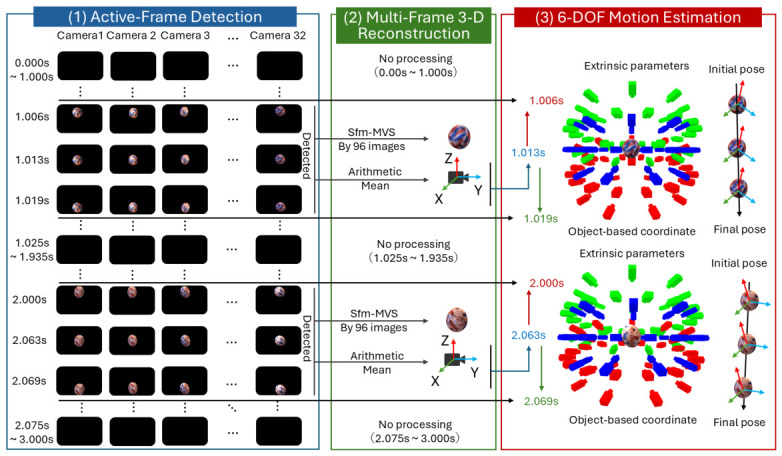
Flow of the free-fall-oriented multi-frame 3-D capture algorithm. (1) Active-frame detection: background subtraction on 32 camera streams identifies frames in which a falling object is present. (2) Multi-frame 3-D reconstruction: the active frames are processed together by SfM–MVS using 96 images (three frames × 32 cameras), followed by arithmetic averaging of per-frame XMP outputs across active frames to obtain 32 stable per-camera extrinsics. (3) Six-DOF motion estimation: the 3-D object position at each frame is recovered by back-projecting silhouette centroids as rays and triangulating their intersection (IRLS), while the orientation is estimated by Procrustes alignment of camera-center shifts across frames (object-based coordinate); kinematic refinement yields the metric 6-DOF trajectory.

On the basis of these considerations, a free-fall-oriented multi-frame 3-D capture algorithm is proposed for small falling objects, integrating rigid-body temporal accumulation with 6-DOF pose estimation. By exploiting temporal redundancy, the algorithm can utilize hundreds of view images for each object, far exceeding the number of physical cameras. The processing flow consists of three main steps: (1) active-frame detection for each falling object, (2) multi-frame 3-D shape reconstruction using SfM-MVS, and (3) 6-DOF motion estimation using SfM-MVS-derived camera parameters. Figure 4 illustrates the overall flow of the algorithm. The details of the algorithm are as follows:

(1) **Active-frame detection for each falling object.**

First, frames in which a falling small object appears are detected by background subtraction. Let $I_{k}^{i}\left(x,y\right)$ denote the image acquired by the *i*-th camera (i=1,…,32) at frame *k*, and let $I_{\mathrm{back}}^{i}\left(x,y\right)$ denote its corresponding background image. For active-frame detection, a representative camera indexed by $i_{a}$ is selected, and the difference image is computed as:

(1) $$D_{k}^{i_{a}}\left(x,y\right)=|I_{k}^{i_{a}}\left(x,y\right)-I_{\mathrm{back}}^{i_{a}}\left(x,y\right)|.$$

If the number of pixels in $D_{k}^{i_{a}}\left(x,y\right)$ whose intensity exceeds a threshold $I_{\theta }$ is greater than $\theta_{0}$, the object is regarded as present in the field of view, assuming that the frames in which the object is visible are identical across all cameras. Frame *k* is then marked as active for object detection with the attribute $a_{k}=1$, and otherwise $a_{k}=0$. Active frames are associated with the same object using a label counter $l_{k}$, updated as

(2) $$l_{k}=l_{k-1}+a_{k-1}\cdot \left(1-a_{k}\right),$$

with $l_{0}=0$. When $a_{k-1}=1$ and $a_{k}=0$, the counter increments, indicating the end of a trajectory. Thus, consecutive frames with $a_{k}=1$ share the same label $l_{k}$, yielding several hundred active frames per object across the 32 cameras and providing sufficient redundancy for robust reconstruction.

(2) **Multi-frame 3-D shape reconstruction using SfM-MVS.**

The active frames obtained in step (1) are grouped by the label counter $l_{k}$. With *L* labels corresponding to *L* falling objects, all frames $\left\{I_{k}^{i}\right\}$ sharing $l_{k}=m$(m=1,…,L) are treated as object *m* and jointly used for 3-D shape reconstruction. For the *m*-th object, let $F^{m}=\left\{k|l_{k}=m\right\}$ be the set of active frames, and $x_{j,k}^{i}$ the 2-D projection of the *j*-th 3-D point $X_{j}^{m}$(j=1,…,J) in camera *i* at frame $k\in F^{m}$, where $X_{j}^{m}$ is an unknown jointly estimated for the *m*-th falling object with the camera parameters in SfM. The perspective projection of $X_{j}^{m}$ is given by

(3) $$s_{j,k}^{i}\;x_{j,k}^{i}=K^{i}\left(R_{k}^{i}X_{j}^{m}+t_{k}^{i}\right),$$

where $K^{i}$ is the intrinsic matrix, $\left(R_{k}^{i},t_{k}^{i}\right)$ the extrinsic parameters of camera *i*, with rotation matrix $R_{k}^{i}$, denoting its orientation and $t_{k}^{i}$ its position in the object-based coordinate system, and $s_{j,k}^{i}$ is a depth scale factor mapping $x_{j,k}^{i}$ to pixel coordinates under the pinhole model. SfM estimates the extrinsic parameters $\left\{R_{k}^{i},t_{k}^{i}\right\}$ of all cameras (i=1,…,32) together with the sparse 3-D structure $\left\{X_{j}^{m}\right\}$, and MVS is then applied to densify the reconstruction. The sparse structure and camera poses are estimated by minimizing the reprojection error over all active frames in $F^{m}$:

(4) $$\min_{\left\{R_{k}^{i},t_{k}^{i},X_{j}^{m}\right\}}\sum_{k\in F_{m}}\sum_{i=1}^{N}\sum_{j=1}^{M}{∥x_{j,k}^{i}-\pi \;\left(K^{i}\left(R_{k}^{i}X_{j}^{m}+t_{k}^{i}\right)\right)∥}^{2},$$

where π(·) is the perspective projection. Thus, for each label *m*, SfM–MVS provides (i) a dense 3-D reconstruction of the object and (ii) the camera extrinsic parameters $\left(R_{k}^{i},t_{k}^{i}\right)$ for all cameras, expressed in the object-based coordinate system, which are then used for pose estimation in step (3). In this study, SfM–MVS processing was performed using a commercial SfM–MVS software package [51]. Specifically, all reconstructions were performed with RealityCapture version 1.4.1 (Build 117424; Capturing Reality/Epic Games), running on workstation PC0 (Intel Core i9-12900KF, 128 GB RAM, NVIDIA GeForce RTX 3090; see Table 1). The default high-quality alignment and reconstruction presets were used unless otherwise stated, and the same preset was applied to every accumulation level so that the reconstructions reported in Section 4 differ only in the input image set, not in the pipeline configuration. It should be emphasized that the SfM–MVS pipeline takes only the multi-view images as input: no prior camera poses, pre-calibrated extrinsics, or external metric scale are provided, and the reconstruction is derived entirely by the software’s internal algorithms. The per-camera extrinsic parameters $\left(R_{k}^{i},t_{k}^{i}\right)$ exported by the SfM–MVS software are, however, reused in step (3) for 6-DOF motion estimation, where they are first stabilized by arithmetic averaging across active frames before being applied to recover the object trajectory. For one-by-one falling objects, efficiency is improved by using only active frames. With $N_{\mathrm{total}}$ total frames and $N_{\mathrm{active}}=\left|F^{m}\right|$ active ones, the reduction ratio $\eta =N_{\mathrm{active}}/N_{\mathrm{total}}$ is typically much smaller than 1 when falling objects appear intermittently, reducing the processing cost to about η times that of conventional volumetric capture.

(3) **Six-DOF pose estimation with kinematic refinement**

The camera extrinsic parameters $\left\{R_{k}^{i},t_{k}^{i}\right\}$ estimated by SfM in step (2) are expressed in the object-based coordinate system and are used to recover the full 6-DOF trajectory of the falling object: its three-dimensional position $P_{k}\in R^{3}$ and orientation $\Phi_{k}\in \mathrm{SO}\left(3\right)$ at each active frame *k*.

Position estimation by multi-view ray triangulation.For camera *i* at frame *k*, the camera center in the world coordinate system is $C^{i}=-{\left(R_{\mathrm{ref}}^{i}\right)}^{⊤}t_{\mathrm{ref}}^{i}$, computed from the reference extrinsics $\left(R_{\mathrm{ref}}^{i},t_{\mathrm{ref}}^{i}\right)$ estimated by the SfM-MVS pipeline. The silhouette of the falling object is detected in image *i* at frame *k* by Otsu thresholding [52], followed by morphological refinement, and its centroid ${\hat{x}}_{k}^{i}$ is estimated robustly as the median of the moment-based centroid, bounding-box center, and fitted ellipse center of the largest contour. A unit viewing ray is then back-projected as
  (5) $$d_{k}^{i}=\frac{{\left(R_{\mathrm{ref}}^{i}\right)}^{⊤}K_{i}^{-1}{\hat{x}}_{k}^{i}}{∥{\left(R_{\mathrm{ref}}^{i}\right)}^{⊤}K_{i}^{-1}{\hat{x}}_{k}^{i}∥},$$
  where $K_{i}$ is the intrinsic matrix of camera *i*. Given rays from all $N_{k}$ cameras at frame *k*, the 3-D object center $P_{k}$ is estimated by minimizing the sum of squared point-to-ray distances:
  (6) $$P_{k}=\underset{X}{arg min}\sum_{i=1}^{N_{k}}w_{k}^{i}{∥\left(I-d_{k}^{i}{d_{k}^{i}}^{⊤}\right)\left(X-C^{i}\right)∥}^{2},$$
  where *I* is the 3×3 identity matrix and $w_{k}^{i}$ denotes the per-ray weight. This weighted linear system is solved in closed form, and the weights are updated iteratively using the IRLS (iteratively reweighted least squares) scheme with a Huber-type kernel to suppress outlier rays caused by segmentation noise or partial occlusion.
Orientation estimation by cross-frame Procrustes alignment. The inter-frame object rotation is estimated by comparing SfM-estimated camera positions across frames. For each frame *k*, the SfM–MVS software exports the camera centers ${\left\{C_{k}^{i}\right\}}_{i=1}^{N}$ in the object-based coordinate system. Because the camera array is physically fixed in the world, any change in the SfM-estimated camera positions between frames is solely due to the rigid-body rotation of the object itself. The inter-frame rotation $\Phi_{k}$ is therefore recovered as the rotation component of the similarity transform that maps $\left\{C_{k}^{i}\right\}$ onto the reference-frame set $\left\{C_{\mathrm{ref}}^{i}\right\}$:
  (7) $$\left(\Phi_{k},\;t_{k},\;s_{k}\right)=\underset{R\in \mathrm{SO}(3),\;t,\;s}{\arg\min}\sum_{i\in I_{k}}{∥C_{\mathrm{ref}}^{i}-sRC_{k}^{i}-t∥}^{2},$$
  where $I_{k}$ is the set of inlier cameras at frame *k*. The problem is solved in closed form via singular value decomposition (SVD) applied to the centered cross-covariance matrix $H=\sum_{i\in I_{k}}\left(C_{k}^{i}-{\bar{C}}_{k}\right){\left(C_{\mathrm{ref}}^{i}-{\bar{C}}_{\mathrm{ref}}\right)}^{⊤}$. Cameras whose residual exceeds twice the median are iteratively rejected as outliers, with a minimum of six inliers enforced, to suppress cameras affected by SfM drift or keypoint mismatches. The rotation component $\Phi_{k}$ gives the estimated inter-frame object orientation.
Metric scale calibration via ICP alignment to a reference 3-D model. Although the camera intrinsics, the per-camera extrinsics, and the sparse 3-D structure are jointly estimated from the multi-view images alone, the reconstruction is inherently up-to-scale: even when the per-camera intrinsics are accurately recovered, and the physical pixel pitch of the sensor (3.45 μm) is known, the global metric scale of the reconstructed scene remains a free degree of freedom of the bundle-adjustment problem and cannot be resolved from imagery alone. Knowing the pixel pitch constrains only the per-pixel angular resolution of each camera; it does not constrain the absolute baseline length between camera centers, which is what fixes the scene scale. Recovering metric scale, therefore, requires an external length reference, supplied here by the ATOS reference mesh. The SfM reconstruction produces positions in an arbitrary coordinate system whose metric scale is unknown. To recover the absolute scale without circular reasoning, the metric scale factor α (mm per SfM unit) is determined independently by aligning the reconstructed 3-D model to a reference 3-D model. In this study, the reference model is acquired with an industrial structured-light scanner (ATOS Compact Scan, Carl Zeiss GOM Metrology, Braunschweig, Germany). The SfM mesh is aligned to the reference 3-D model mesh using multi-start Iterative Closest Point (ICP) [53], followed by point-to-plane refinement, and the scale factor is computed as
  (8) $$\alpha =\frac{d_{\mathrm{ref}}}{d_{\mathrm{SfM}}},$$
  where $d_{\mathrm{ref}}$ and $d_{\mathrm{SfM}}$ denote the maximum bounding-box extents of the reference and the SfM model, respectively. The multi-start ICP uses only geometric (point-to-point and point-to-plane) correspondences; texture and color are not incorporated into the registration objective, since the ATOS reference mesh is purely geometric and a color-based residual would be ill-defined on the reference side. Even if a textured reference were hypothetically available, photometric consistency could not be guaranteed across two independent acquisitions performed by different instruments under different illumination, white-balance, and sensor-response settings, and any color-based residual would therefore remain unreliable. The raw position estimates are then converted to millimeters as ${\tilde{P}}_{k}=\alpha \;\left(P_{k}-P_{1}\right)$.

## 4. Experiments

**Figure 5:**
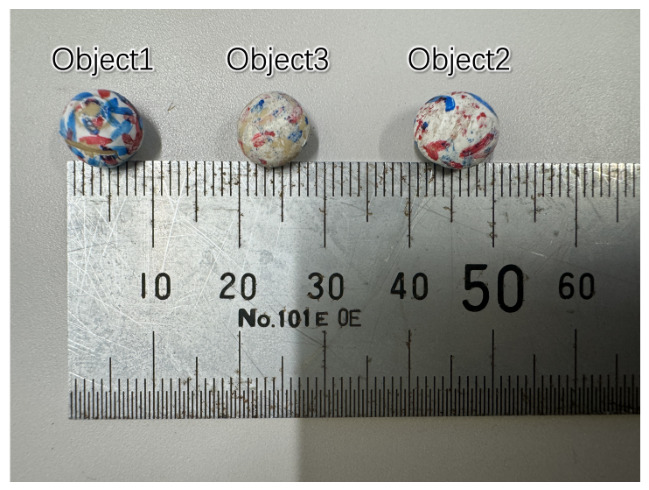
The three soybean specimens used as test objects in all experiments (Object 1, Object 2, and Object 3, approximately 8–10 mm in diameter).

The experiments are organized in three parts. Section 4.1 validates the multi-frame accumulation strategy by quantifying reconstruction quality as a function of the cumulative number of input images. Section 4.2 evaluates the geometric accuracy and repeatability of the resulting 3-D models across 30 independent free-fall trials against an industrial reference scanner, and demonstrates that fine surface features such as structural cracks are preserved in the reconstruction. Section 4.3 demonstrates the full 6-DOF kinematic analysis capability, illustrating how shape and motion information are recovered simultaneously from a single free-fall sequence. In all experiments, soybeans of approximately 8–10 mm diameter were used as test objects. Figure 5 shows the three soybean specimens used throughout the experiments.

### 4.1. Multi-Frame Accumulation and Reconstruction Quality

The proposed algorithm accumulates images from multiple consecutive frames into a single SfM-MVS reconstruction, effectively increasing the number of viewpoints beyond the physical 32 cameras. To characterize how reconstruction quality evolves with the number of input images, progressive accumulation experiments were conducted on two objects: Object 1 (approximately 9 mm diameter) and Object 2 (approximately 10 mm diameter), each during a single free-fall trial. Starting from frame *K* in which the object is fully visible, eight images were selected from the spatially nearest camera cluster (identified from the SfM-MVS-estimated camera positions stored in XMP metadata, exported by the SfM-MVS software after a preliminary reconstruction). Additional images were then cumulatively added in groups of eight by expanding outward from frame *K* in a K±1,K±2,… sequence—rather than accumulating chronologically from the first to the last frame in which the object appears—so that the base reconstruction is built from the highest-quality observations and temporally distant frames (which may contain truncated views near the entry and exit of the field of view) are added last. An independent SfM-MVS reconstruction was performed at each accumulation level, yielding 24 incremental models from 8 to 192 images per object. Because the 32 physical cameras capture one frame simultaneously, 32 images correspond to a single frame, 64 images to two accumulated frames, and 96 images to three accumulated frames.

Prior to error evaluation, each reconstructed mesh was denoised by removing disconnected triangle clusters containing fewer than 1% of the total triangle count. This eliminates small floating artifacts produced by the SfM-MVS pipeline while preserving all substantial body surfaces. Each cleaned mesh was then aligned to a reference 3-D model obtained by an industrial 3-D scanner (ATOS Compact Scan) via the following procedure. A fixed scale factor was determined for Object 1 and Object 2, respectively, by computing the bounding-box extent ratio between the final (192-image) denoised model and the reference. Each scaled model was uniformly sampled to $1\times 10^{5}$ points, centroid-aligned to the reference, and refined by multi-start ICP using 64 initial rotations. The best candidate from coarse point-to-point ICP was further refined by point-to-plane ICP for up to 2000 iterations. In cases where the ICP-aligned result exhibited a higher root mean square error (RMSE) than the centroid-only alignment, the centroid-only alignment was retained as a fallback. After alignment, the one-directional Chamfer distance from the reconstructed 3-D model to the reference model was computed, and four metrics were derived: mean error, RMSE, maximum error, and F-score at a 0.5 mm threshold, corresponding to approximately 5% of the object diameter. The F-score was defined as the harmonic mean of precision and recall at this threshold.

**Figure 6:**
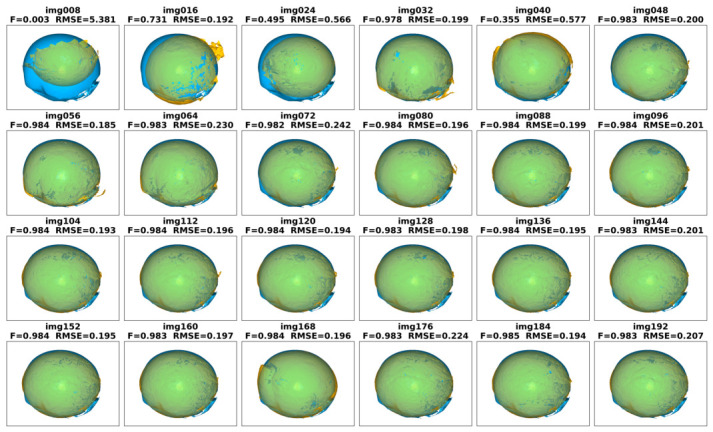
Progressive reconstruction quality for Object 1 with increasing number of accumulated images, shown as front-view overlays against the ATOS reference. Yellow: proposed reconstruction; cyan: ATOS reference; green regions indicate overlap where the two surfaces coincide. Each panel indicates the model name, F-score, and RMSE.

**Figure 7:**
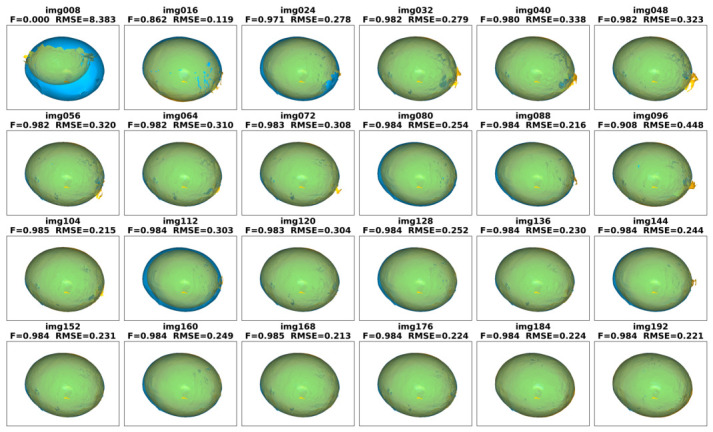
Progressive reconstruction quality for Object 2, presented in the same format as Figure 6.

Figure 6 and Figure 7 show the front-view overlays of all 24 incremental models (yellow) against the ATOS reference (cyan) for Object 1 and Object 2, respectively, with the F-score and RMSE indicated below each panel. For both objects, the eight-image reconstruction covers only a small fraction of the object surface, leaving large areas of the reference exposed. By 32 images (one complete frame), the reconstructed surface closely conforms to the ATOS reference, and from 48 images onward, the two meshes are visually nearly indistinguishable.

Table 2 quantifies the reconstruction quality for Object 1. With only eight images, the model is highly incomplete (F = 0.004). A sharp transition occurs at 32 images (one complete frame), where the F-score jumps to 0.978, and the RMSE drops to 0.199 mm. Beyond 48 images, the metrics plateau at F ≈ 0.984 with RMSE ≈ 0.19 mm.

The same convergence pattern is observed for Object 2 (Table 3): the F-score reaches 0.982 at one frame and generally stabilizes at F ≈ 0.984 with RMSE ≈ 0.22–0.25 mm. An isolated dip to F = 0.908 at 96 images is attributable to ICP alignment converging to a local minimum for that particular model; the metrics recover immediately at 128 images, closely matching the convergence behavior of Object 1.

The maximum error stabilizes near 1.6 mm for both objects once 24 or more images are used; this residual mainly arises from the mounting-rod occlusion region in the ATOS reference rather than from a reconstruction deficiency, whereas the proposed free-fall system provides background-free 3-D models of unsupported mid-air objects.

These results confirm that the temporal integration strategy effectively compensates for the limited number of physical cameras. Since reconstruction quality plateaus beyond approximately one to two frames, the subsequent experiments use all detected active frames (typically 5–6 frames) to provide redundant viewpoints, improving the robustness of matching and the completeness of the reconstruction.

### 4.2. 3-D Shape Fidelity Evaluation

To evaluate both the robustness and the geometric accuracy of the proposed system, a total of 30 independent free-fall trials were conducted using three soybean objects (10 trials per object), and the resulting reconstructions were compared against reference 3-D models acquired with the ATOS Compact Scan.

**Figure 8:**
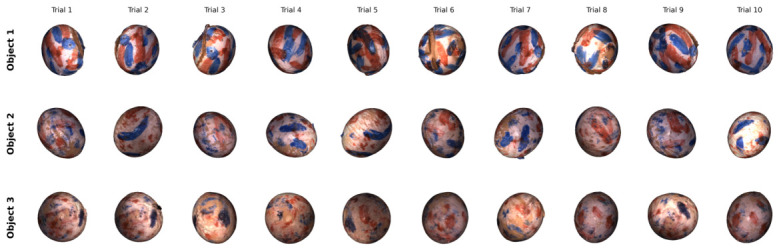
Textured 3-D models from 30 independent free-fall trials (3 objects × 10 trials), each rendered from a randomly sampled viewpoint. Each model was reconstructed using the proposed multi-frame SfM-MVS pipeline with all detected active frames (typically 5–6 frames, approximately 170–200 images). The consistent shape and texture reproduction across all 30 trials demonstrates the system’s robustness and repeatability under varied fall conditions.

Figure 8 presents the textured 3-D models reconstructed from all 30 trials, each rendered from a randomly sampled viewpoint to highlight surface texture diversity. Despite variations in initial pose, release angle, and tumbling dynamics across different trials, the system consistently produces complete, well-formed 3-D models with recognizable surface texture patterns. The visual consistency across all 30 trials demonstrates that the proposed capture and reconstruction pipeline is robust to the stochastic nature of free-fall motion and can reliably operate in a high-throughput inspection scenario.

The reconstructed mesh was uniformly sampled and aligned to the reference 3-D model using the same multi-start ICP procedure described in Section 4.1. The mean error, RMSE, and maximum error were computed from the one-directional Chamfer distance.

**Figure 9:**
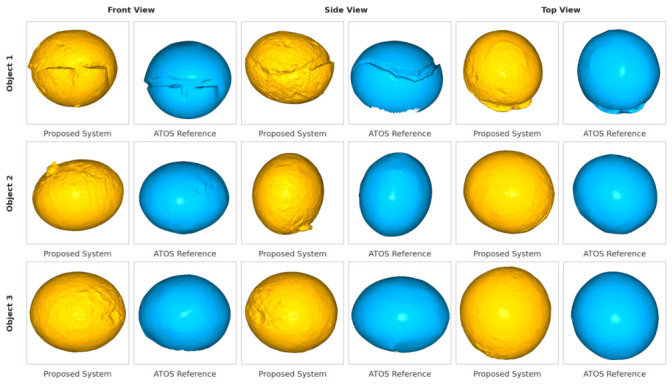
Multi-view visual comparison of representative 3-D models for three soybean objects, shown from front, side, and top viewpoints. In each pair, the left model (yellow) was 3-D reconstructed by the proposed system during free fall, and the right model (cyan) is the reference 3-D model acquired by an ATOS Compact Scan under static conditions.

Figure 9 presents a representative multi-view visual comparison of the 3-D reconstructed models (yellow) and the reference 3-D models (cyan) for each of the three objects from front, side, and top viewpoints. Each displayed model corresponds to the trial whose RMSE is closest to the median value among the 10 trials for that object. The overall geometric contours reconstructed by the proposed system closely match the ATOS references across all three viewpoints, confirming that the proposed free-fall capture pipeline preserves the global shape of the target objects despite the challenges of high-speed dynamic imaging.

Table 4 summarizes the shape fidelity error statistics across all 30 trials. The overall mean error is 0.146±0.033 mm and the overall RMSE is 0.235±0.043 mm, with an overall F-score of 0.980±0.006 at the 0.5 mm threshold, demonstrating sub-millimeter accuracy on objects approximately 8–10 mm in diameter (relative error ≈ 1.6%). Object 1 and Object 2 achieve comparable accuracy (F-score 0.984 and 0.985, respectively), while Object 3 exhibits a slightly lower F-score (0.971±0.001). The standard deviations across all metrics are small relative to the mean values, indicating that the proposed system produces geometrically consistent reconstructions across repeated free-fall trials.

No severe outliers are present in any of the three metrics, indicating that the system does not suffer from occasional reconstruction failures. The maximum errors of approximately 1.6–2.1 mm are localized at boundary regions where partial occlusion or sparse texture reduces the reconstruction fidelity, and do not represent the overall surface quality. Although the absolute precision does not reach the level of a dedicated structured-light scanner such as the ATOS Compact Scan (typical accuracy ∼0.01 mm), the proposed system achieves sub-millimeter shape accuracy under fully dynamic, non-contact, high-throughput conditions—a regime in which conventional industrial scanners cannot operate.

**Figure 10:**
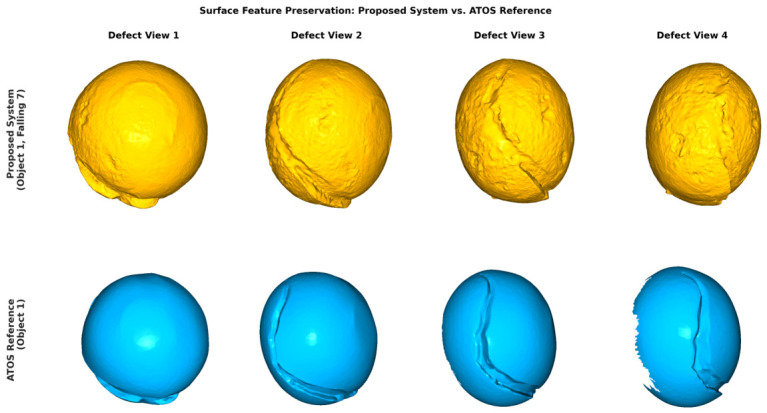
Surface feature preservation: selected views of Object 1 reconstructed during free fall (**top row**, yellow) and the reference 3-D model captured by ATOS Compact Scan (**bottom row**, cyan). The prominent structural crack running across the equatorial region is consistently reproduced in the proposed system’s reconstruction, demonstrating that reconstruction fidelity is sufficient to resolve sub-millimeter surface defects.

Beyond global shape accuracy, the reconstructed models also preserve fine surface features that are of direct relevance to quality inspection. Figure 10 presents selected views of Object 1 from a free-fall trial (Trial 7) alongside the corresponding reference 3-D model captured by ATOS Compact Scan, both revealing a prominent structural crack running across the equatorial region of the soybean. The crack is consistently reproduced in the proposed system’s reconstruction with correct geometry and position, demonstrating that reconstruction fidelity is sufficient to resolve sub-millimeter surface defects. This level of geometric preservation provides a foundation for downstream automated defect detection algorithms—for example, local curvature analysis or template-based anomaly detection—which is significant for agricultural quality control, where surface cracks and dents are the primary indicators of seed damage and reduced viability.

### 4.3. Six-DOF Kinematic Analysis of Free-Falling Objects

Building on the reconstruction quality validated in Section 4.1 and Section 4.2, we demonstrate the system’s ability to simultaneously capture shape evolution and full kinematic state from a single free-fall sequence. A six-degree-of-freedom (6-DOF) kinematic analysis was conducted on two freely falling soybeans; the same 160 fps videos were used to analyze Trial 2 of Object 1 and Trial 9 of Object 2 in Section 4.2. The active frames were selected for pose estimation: five frames (Frames 26–30) for Object 1, and six frames (Frames 52–57) for Object 2.

The calibrated position trajectory is validated by fitting an unconstrained quadratic polynomial $P\left(t\right)=P_{0}+V_{0}t+\frac{1}{2}a\;t^{2}$ and comparing the fitted acceleration magnitude ∥a∥ to the gravitational constant g=9807 mm/s^2^ as an independent consistency check. The smoothed positions ${\hat{P}}_{k}=P_{0}+V_{0}t_{k}+\frac{1}{2}a\;t_{k}^{2}$ from this fit are used for the trajectory visualizations below.

For orientation, the raw rotations $\left\{\Phi_{k}\right\}$ are smoothed under a constant angular velocity assumption. Each rotation is mapped to its SO(3) logarithm ${\tilde{\omega }}_{k}=\log\left(\Phi_{k}\right)$, and a single angular velocity ω is estimated by linear regression:

(9) $$\omega =\underset{\omega }{\arg\min}\sum_{k}{∥{\tilde{\omega }}_{k}-\omega \;t_{k}∥}^{2},$$

from which the smoothed orientations are reconstructed as ${\hat{\Phi }}_{k}=\exp\left(\omega \;t_{k}\right)$. The smoothed orientations are expressed relative to the first frame as ${\tilde{\Phi }}_{k}={\hat{\Phi }}_{k}\;{\hat{\Phi }}_{1}^{⊤}$. The resulting trajectory $\left\{\left({\tilde{P}}_{k},\;{\tilde{\Phi }}_{k}\right)\right\}$ provides the complete metric position and orientation history of the falling object at each active frame.

Object 1 yielded ∥a∥=9967 mm/s^2^ (+1.6% deviation from *g*), and Object 2 yielded ∥a∥=9764 mm/s^2^ (−0.4% deviation), confirming the accuracy of the ICP-based scale calibration. The initial velocities were estimated at $|V_{0}|=0.61$ m/s for Object 1 and $|V_{0}|=0.34$ m/s for Object 2. Because each object was manually released from a fixed support above the camera array, the release height varied slightly between trials. Accordingly, $|V_{0}|$ denotes the velocity at the first measured active frame rather than at the instant of release, and the difference between the two objects simply reflects the free-fall distance traversed before each object entered the measurement volume; it is not a property of the measurement system.

**Figure 11:**
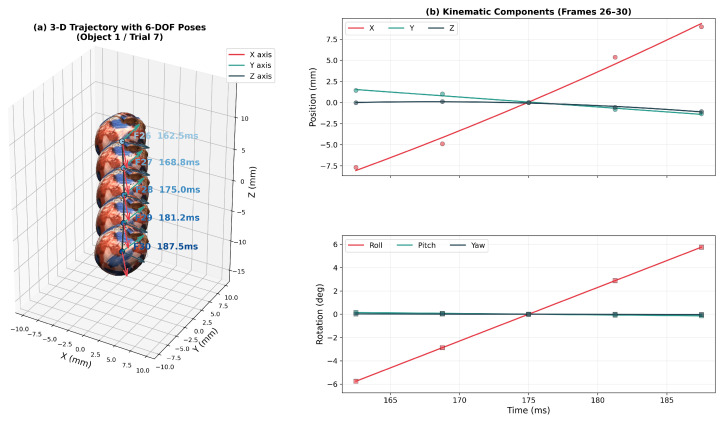
Six-DOF kinematic analysis of Object 1 (9 mm soybean, Frames 26–30): (**left**) 3-D trajectory with body-frame orientation arrows and textured model at each node; (**right**) kinematic components versus time, showing translation (**top**) and Euler angles (**bottom**).

**Figure 12:**
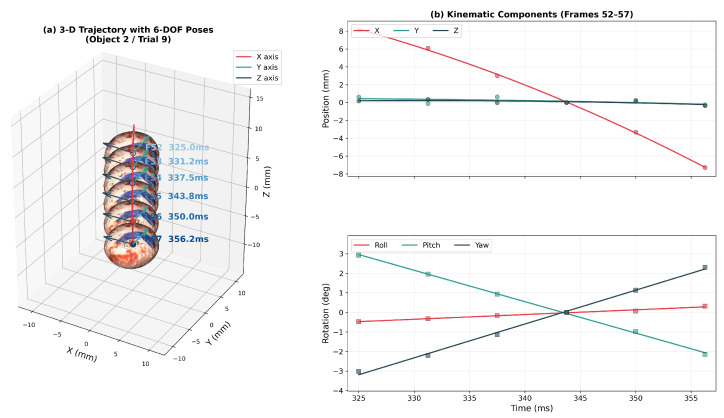
Six-DOF kinematic analysis of Object 2 (10 mm soybean, Frames 52–57): (**left**) 3-D trajectory with body-frame orientation arrows and textured model at each node; (**right**) kinematic components versus time.

Figure 11 and Figure 12 present the reconstructed 6-DOF motion for each object. The left panels show the falling trajectories with body-frame orientation arrows and the textured 3-D model embedded at each trajectory node, illustrating the spatial pose of the object at each instant. The right panels present the kinematic components over the measured frames: the position curves show the expected parabolic separation under gravitational acceleration, and the Euler angle curves, smoothed under the constant angular velocity model, are consistent with stable rotation during free fall. The angular velocities were estimated at approximately 460°/s (1.3 rev/s) for Object 1 and 237°/s (0.7 rev/s) for Object 2. The lateral deviation from a straight-line trajectory was 0.53 mm for Object 1 and 0.13 mm for Object 2, confirming negligible aerodynamic drift. These results demonstrate that the proposed system can reliably extract both the evolving 3-D geometry and the complete dynamic state of small freely falling objects.

## 5. Discussion

### 5.1. Computational Cost and Path to High-Throughput Processing

The proposed system separates the measurement pipeline into two stages: real-time data acquisition and offline reconstruction. Active-frame detection relies on background subtraction and is computationally negligible. The SfM-MVS reconstruction, performed using the SfM-MVS software [51], is the computational bottleneck. In practice, processing one object (three accumulated frames, 96 images total) requires approximately 6 s for camera alignment, 27 s for dense reconstruction, and 17 s for texturing—a total of roughly 50 s on a laptop GPU (NVIDIA GeForce RTX 4090) and approximately 1 min on the laboratory workstation (PC0, NVIDIA GeForce RTX 3090). Although this precludes true real-time throughput at present, the temporal sparsity of the free-fall sequence provides a meaningful efficiency advantage. With a total recording window of 7 s at 160 fps, the ratio of active to total frames is typically 0.04375, reducing the SfM-MVS input by a factor of 22.86 compared to processing all frames, as discussed in Section 3. Furthermore, the PC cluster architecture allows reconstruction of successive objects to be pipelined across multiple nodes in parallel, partially decoupling per-object latency from overall throughput. A clear path toward real-time operation exists: replacing the SfM-MVS software with a lightweight GPU-accelerated pipeline—such as a custom COLMAP-based incremental SfM combined with a learning-based MVS depth estimator—would eliminate software overhead and reduce per-object reconstruction time to seconds. This represents the primary direction for future engineering work.

The processing time (s) column added to Table 2 and Table 3 characterizes the per-step wall-clock cost of the SfM–MVS pipeline as a function of the cumulative input image count on PC0. Across both objects, the per-step time grows roughly linearly with the number of input images, from approximately 13–25 s at the first eight-image step to 85–160 s above the 150-image level, and remains bounded out to 200 images. Object 1 completes the 24-step accumulation in 29.6 min in total, and Object 2 completes the 25-step accumulation in 32.3 min in total (single run for each). The workstation-level totals are therefore consistent between the two objects. The per-step times include some run-to-run non-determinism inherent to the SfM–MVS pipeline, originating in feature matching, bundle adjustment, and dense matching. Together with the rapid reconstruction-quality plateau reported in Section 4.1, where the F-score saturates after one to two accumulated frames, only a small number of additional frames need to be processed in practice to reach near-saturation accuracy, and the per-step compute cost stays bounded as additional temporal frames are added.

### 5.2. Texture Dependency and Failure Modes

The proposed reconstruction pipeline relies on SfM feature matching and is therefore inherently dependent on the presence of distinctive surface texture. Experiments with textureless objects such as white rice grains resulted in complete reconstruction failure, as no stable keypoint correspondences could be established across views. Objects with partially specular surfaces—such as polished cylindrical plastic beads bearing a printed character—behaved differently: the character provided sufficient texture anchors for SfM to succeed, but reflective surface regions introduced view-dependent brightness variations that occasionally produced local reconstruction artifacts. These observations define the operating regime of the current system: objects must exhibit spatially varied, diffuse surface appearance to support reliable reconstruction. For objects that fall outside this regime, two complementary strategies are applicable. First, active texture projection using a structured-light pattern illuminator co-mounted with the camera array could impose artificial features on featureless surfaces without modifying the object. Second, learning-based reconstruction methods such as NeRF [17] or 3D Gaussian Splatting [23] can potentially recover geometry from appearance cues alone, though they currently require per-scene training that is difficult to apply in a high-throughput setting.

**Figure 13:**
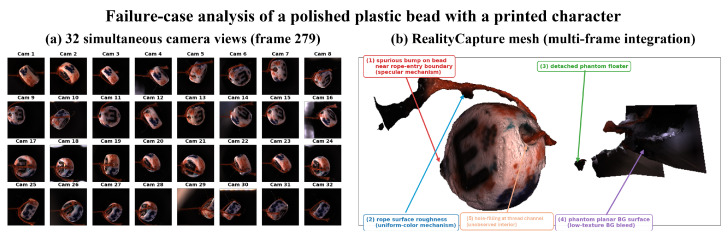
Failure-case analysis of a polished plastic bead bearing a printed character (‘E’), suspended on red thread for stationary characterization. (**a**) All 32 synchronized cameras viewing the bead at the same instant (frame 279); view-dependent specular highlights are concentrated near the silhouette edges and shift to a different edge region of the bead in each view. (**b**) RealityCapture mesh built from 640 input images, of which 468 were successfully aligned (multi-frame integration across many time instants). Five characteristic late-stage reconstruction artefact mechanisms are annotated and discussed in the text: (1) spurious bumps near the silhouette regions of the bead; (2) bumps along the rope; (3) detached phantom floaters; (4) phantom planar background surfaces; and (5) geometric hole-filling at the bead’s central thread channel.

To make the late-stage failure modes concrete, Figure 13 reports a representative reconstruction of a polished plastic bead bearing a printed character (’E’, present on both faces) suspended on a uniform red thread for stationary characterization; the bead surface combines diffuse painted features (the character, which anchors SfM correspondences) with polished specular regions (the surrounding bead body). Panel (a) shows all 32 cameras viewing the bead at the same instant (frame 279, all cameras hardware-triggered simultaneously): specular highlights are concentrated near the bead’s silhouette edges, where the local surface normal is most sensitive to viewing direction, and the highlight shifts to a different edge region of the bead in each view. Panel (b) shows the resulting RealityCapture mesh, built from 640 input images of which 468 (73 %) were successfully aligned, i.e., a multi-frame integration across many time instants rather than the single-instant 32-camera set of panel (a). Five characteristic late-stage reconstruction artifact mechanisms are visible, all of which share a common root cause—SfM/MVS is misled wherever reliable texture is unavailable—and produce, for example:

1. Spurious bumps on the bead body, near the silhouette regions of the object in each image. View-dependent specular highlights affect SfM feature matching in two complementary ways: (a) they act as spurious anchors—their apparent position differs between viewpoints, so the back-projected geometry contains small protrusions in the highlighted regions; and (b) reflected light occludes the genuine anchors on which SfM relies—namely the diffuse painted texture on the bead surface—as visible in the per-camera time series for this dataset, where the mobile highlight progressively covers different regions of the painted texture as the bead rotates, depriving SfM of stable correspondences exactly where they would otherwise be most reliable.
2. Bumps along the rope itself. The thread’s nearly uniform red color provides limited local feature variation, so MVS depth estimation is under-constrained along the rope’s length and the reconstructed rope contains small deviations instead of being smooth.
3. Detached phantom floaters—small disconnected mesh fragments—produced by mismatched correspondences between uniformly black background pixels across different cameras.
4. Phantom planar surfaces with dark texture adjacent to the rope, produced where the rope’s uniform color and the dark background leave the depth estimator without a sharp transition; depth bleeds into the background and bakes out as a flat sheet colored by the background.
5. Geometric hole-filling artifact at the bead’s central thread channel. The bead has a real through-hole at its center to allow the suspension thread to pass through it; on the face shown in panel (b), this hole has been closed up in the reconstruction. The mechanism is not specular: the channel inner walls bear no painted texture, MVS therefore receives no depth signal for that region, and the surface-completion stage closes the open boundary by smoothness default. The same mechanism would cause the system to under-report holes, slots, or recessed features in industrial parts whose interior surfaces are not directly viewable.

Mechanisms (2) and (4) are perturbations specific to the bead’s suspension setup; in the actual industrial deployment scenario, where free-falling objects are imaged in continuous flow without any suspension thread, they do not arise. Mechanisms (1) and (5), namely the bead-surface specular component and the unobserved-interior closure, would persist for highly polished or perforated targets and constitute the principal regimes in which the proposed system is currently vulnerable. Mechanism (3) can also persist in the free-fall deployment scenario; the redundant images provided by multi-frame integration are relied upon to suppress it. On the hardware side, replacing the present direct LED illumination with a diffuse source, such as a light tent, a ring softbox, or a partial dome enclosure, could substantially attenuate the view-dependent specular highlights identified in mechanism (1). This is included as a future-work direction for polished-target reconstruction.

A second deployment regime that warrants explicit characterization is high object speed, such as scenarios in which objects are dropped from a significant height or blown along an air stream. With sufficient illumination, the reconstruction does not have a hard physical speed ceiling; the achievable speed is set by the acquisition parameters.

Two effects bound the achievable speed. The exposure must be short enough to freeze the object’s motion within a single frame; otherwise, the image blurs and SfM feature extraction becomes unreliable. The frame rate must, in turn, be high enough that the object does not displace too far between consecutive frames; otherwise, inter-frame rigid-body alignment fails. Both bounds scale linearly with the acquisition parameters, so faster objects can be accommodated by shortening the exposure and raising the frame rate. The accompanying cost is that brighter illumination is required to compensate for the shorter exposure.

The two bounds can be evaluated at the system’s current acquisition settings: frame rate 160 fps, exposure $T_{\exp}=1/9009$ s ≈111μs, ground sampling distance GSD ≈13μm/pixel, and target diameter d≈10 mm. The motion-blur bound is $v_{\mathrm{blur}}=n_{\mathrm{tol}}\cdot \mathrm{GSD}/T_{\exp}$, evaluating to about 0.12, 0.35, and 0.59 m/s for tolerated blur extents of one, three, and five pixels, respectively. The inter-frame displacement bound is $v_{\mathrm{disp}}≲\alpha \cdot d\cdot \mathrm{fps}$, evaluating to about 0.16, 0.48, and 0.80 m/s for α=0.10,0.30,0.50. Combining the two, the effective upper bound at the present settings is $v_{\max}\approx 0.5$–0.6 m/s, depending on the tolerated motion blur, with motion blur becoming the binding constraint slightly before inter-frame displacement does. The experiments in Section 4 were conducted at object velocities of 0.34–0.61 m/s, which is consistent with this bound.

Because the bounds scale linearly with the acquisition parameters, the operating envelope can be extended directly. Targeting v≈5 m/s, representative of an air-blown particle stream, would require shortening the exposure to about 13 μs and raising the frame rate to about 1500 fps. The latter exceeds the present sensor’s full-frame specification (the DFK 37BUX273 supports up to 236 fps at 1440 × 1080) and would require either a reduced-ROI acquisition mode or a camera with a higher native frame-rate, in addition to a substantially stronger light source. Beyond this envelope, exceeding the bounds first manifests as elongated point clouds along the motion direction, due to motion-blur-induced feature drift, and then as a failure of the rigid-body alignment between frames.

### 5.3. Hardware Scalability and Configuration Trade-Offs

The current three-layer, 32-camera configuration was designed to provide uniform omnidirectional coverage within a compact form factor. The circular ring geometry is inherently modular: additional cameras or layers can be incorporated to further increase viewpoint density, and the ring diameter can be adjusted to accommodate objects of different sizes. A key trade-off exists between camera count and per-camera resolution: a smaller array of higher-resolution cameras could achieve comparable reconstruction quality with reduced hardware cost, provided that the object is large enough to fill a sufficient fraction of the image plane. For sub-centimeter targets such as soybeans, however, the 32-camera configuration with 3.45 μm/pixel resolution is close to the minimum required to resolve surface features at the sub-millimeter level. Scaling to smaller objects would require either a proportionally reduced ring diameter or cameras with higher pixel density.

### 5.4. Empirical Validation of the 8+16+8 Topology via Sub-Sampling Ablation

The 32-camera 8+16+8 three-layer architecture used in this study is the outcome of a progressive system-development process originally guided by qualitative engineering judgment: an 8-camera single-ring configuration was rejected because the 45° inter-camera spacing did not provide enough feature overlap for SfM convergence; a 16-camera single-ring configuration was rejected because the equatorial-only viewpoints could not capture polar regions of the falling object; and a two-layer 16+16 configuration was rejected because the wide inter-layer gap caused the reconstruction to fragment into two disconnected components. While these design choices were validated qualitatively during system development, a systematic quantitative comparison across topology and camera-count variants had not previously been performed.

**Figure 14:**
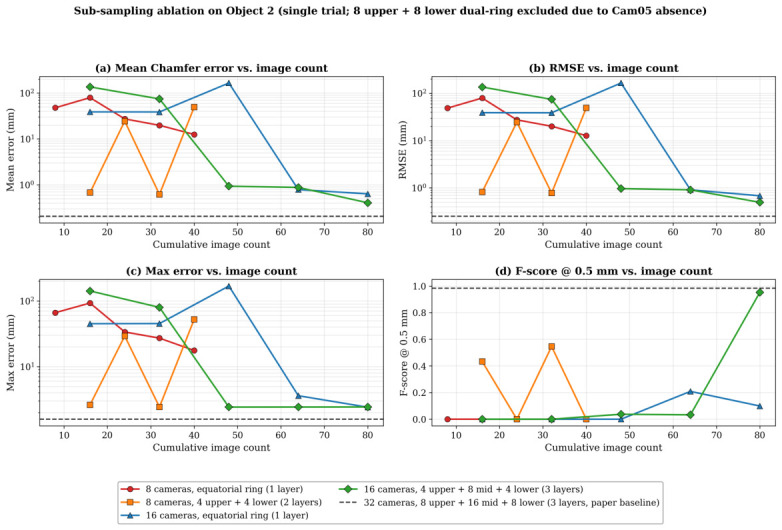
Per-step (**a**) mean Chamfer error, (**b**) RMSE, (**c**) max error, and (**d**) F-score @ 0.5 mm for the four sub-array configurations of Table 5, plotted against the cumulative input-image count. The non-monotonic mid-step behavior visible for the 8-camera dual-layer and the 16-camera single equatorial ring reflects RealityCapture SfM–MVS bundle-adjustment stochasticity near the convergence threshold.

**Figure 15:**
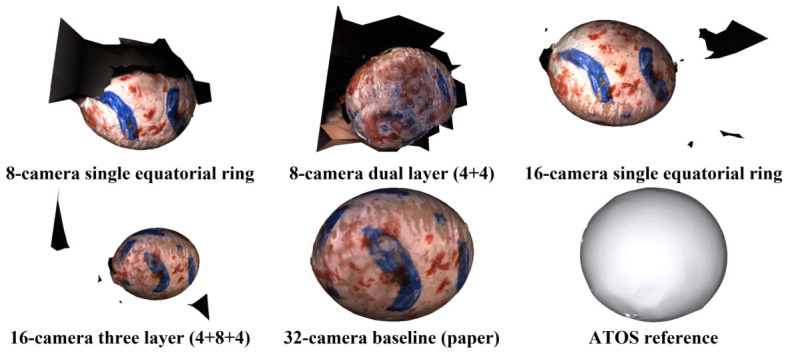
Visual comparison of the final-step reconstruction for each of the four sub-sampling configurations of Table 5, alongside the 32-camera Object 2 baseline and the ATOS reference. The visual ranking, judged by geometric completeness and by the amount of phantom or disconnected debris, agrees with the F-score ranking in Table 5. The 8-camera single-ring fragments into background debris around an incomplete body, the 8-camera dual-layer recovers the gross body shape but misses the polar regions, the 16-camera single-ring is incomplete because of absent polar viewpoints, and the 16-camera three-layer is visually close to the 32-camera baseline. The ATOS reference is shown from a side without point-cloud holes for clean visual comparison.

To quantify these design trade-offs and to assess whether comparable accuracy can be obtained from fewer cameras across additional temporal frames, a sub-sampling ablation was conducted on the existing Object 2 free-fall sequence. From the same 195 captured images, four camera sub-arrays were extracted and processed frame-by-frame through the same SfM–MVS pipeline as the main results. The best-step results (highest F-score per configuration) are summarized in Table 5, the full per-step curves are shown in Figure 14, and a side-by-side visual comparison of the final-step reconstructions is provided in Figure 15.

Three findings emerge from this ablation:

(1) **Under the two tested eight-camera topologies, eight cameras were insufficient to produce a geometrically complete individual model from a single free-fall sequence.** The eight-camera single equatorial ring causes SfM to diverge entirely (F-score = 0 at every accumulation step); the eight-camera dual-layer configuration converges only sporadically, with best F-score = 0.55, far below the sub-millimeter target of the system. Neither eight-camera configuration produces a single-pass, geometrically complete reconstruction of an individual object under either topology.
(2) **Sixteen cameras can produce a reconstruction, but the result depends critically on topology, not on camera count.** The 16-camera single equatorial ring plateaus at F-score = 0.21 with RMSE 0.91 mm; pure azimuthal accumulation cannot substitute for the missing polar viewpoints, regardless of how many cumulative images are processed. By contrast, the 16-camera three-layer configuration reaches F-score = 0.953 with RMSE 0.50 mm, within about 3 % of the 32-camera Object 2 baseline F-score of 0.984.
(3) **The 32-camera 8+16+8 design retains a measurable precision advantage.** RMSE is approximately twice as low as the 16-camera three-layer subset (0.25 versus 0.50 mm), and the F-score margin (0.984 versus 0.953) confirms that a higher fraction of the reconstructed surface lies within the 0.5 mm tolerance. The additional 16 cameras of the published design, therefore, contribute primarily to tighter overall geometric fidelity and to tolerance against single-camera failures, as evidenced by the unrecorded Cam05 in this Object 2 sequence. The Cam05 absence also prevented an 8+8 dual-ring sub-array (eight upper plus eight lower cameras) from being evaluated on equal footing in this sequence, and that scheme is therefore excluded from the ablation table.

Substituting additional temporal frames for additional spatial cameras is, in principle, a sound design substitution. In the present scenario, however, it is imperfect for a quantitative reason. The 6-DOF analysis in Section 4.3 reports object angular velocities of approximately 460°/s for Object 1 and 237°/s for Object 2, which at the 160 fps frame rate correspond to inter-frame rotations of only 2.9° and 1.5° per frame, respectively. Accumulating *N* additional temporal frames therefore adds only of order $3^{\circ }\cdot N$ of azimuthal viewpoint diversity per camera. Replicating the 11.25° angular density of a 32-camera ring starting from an eight-camera ring (45° spacing) would require filling 33.75° intermediate gaps, equivalent to about 12 frames for Object 1 and 23 for Object 2. This is an order of magnitude more than the one-to-two-frame plateau observed with the full 32-camera array.

More fundamentally, free-fall rotation contributes only to azimuthal viewpoint diversity. A free-falling object does not invert vertically, so no temporal frame can provide a top-down or bottom-up view that was not already supplied by the upper and lower eight-camera rings. This is corroborated by the F = 0.21 ceiling of the 16-camera single equatorial ring even at 80 cumulative images. Within the regime studied, that is, free fall with no agitation and no spin-axis change, temporal frames can therefore partially compensate for reduced azimuthal coverage, but cannot replace the polar viewpoints supplied by the upper and lower eight-camera rings.

The ablation establishes a clear qualitative ranking among the tested configurations: single-ring sub-arrays (at any camera count) perform worst, the 8-camera dual-layer is intermediate, and the 16-camera three-layer is close to the full 32-camera baseline. This ranking is sufficient to support the published 32-camera design choice. A more comprehensive ablation across multiple trials, with additional topology variants and dedicated recordings at reduced camera counts, is left as future work. In particular, the 8+8 dual-ring scheme could not be evaluated cleanly here because of the missing Cam05. The data-processing cost mentioned in Section 5.1 itself motivates the camera-constrained regime, since reducing the synchronized camera count proportionally reduces the per-frame data volume.

### 5.5. Industrial Significance of 6-DOF Kinematic Estimation

The simultaneous estimation of 3-D shape and 6-DOF motion from a single free-fall sequence has direct practical consequences for industrial inspection systems. Beyond confirming that an object is falling freely (which validates the rigid-body and sparse-fall assumptions of the algorithm), the recovered trajectory enables the downstream handling equipment to be precisely synchronized with each individual object. Specifically, given the estimated position ${\tilde{P}}_{k}$ and velocity at the last measured frame, the object’s landing position at any subsequent time can be predicted by extrapolating the kinematic model, allowing a pneumatic ejection nozzle or a robotic actuator to be triggered with sub-centimeter spatial accuracy. This capability is particularly relevant for high-speed grain sorting, where defective seeds must be removed from a continuous flow without disrupting the remaining product. The constant angular velocity rotation observed during free fall (Section 4.3) further simplifies this prediction, since no spin-induced drift needs to be compensated. These results suggest that a production-grade system built on the proposed architecture could perform simultaneous non-contact shape inspection and trajectory-based sorting in a single measurement step. More broadly, conventional structured-light scanners such as the ATOS Compact Scan require physical fixturing of each object, placement of background reference markers, and manual removal of background artifacts from the output point cloud; the resulting models are also geometrically incomplete at the mounting interface. These constraints make such instruments fundamentally incompatible with continuous, high-throughput inspection of individually falling objects. The proposed system eliminates all of these requirements: objects fall freely without contact, the reconstruction pipeline automatically isolates each object from the background, and the output is a geometrically complete, fixture-free 3-D model—properties that are indispensable prerequisites for any practical inline inspection pipeline.

## 6. Conclusions

This study proposed a multi-frame temporal integration approach for 3-D shape measurement and 6-DOF kinematic analysis of small freely falling objects. By exploiting the rigid-body assumption, images captured at different time instants by a 32-camera array operating at 160 fps are accumulated into a single SfM-MVS reconstruction, effectively multiplying the number of viewpoints beyond the physical camera count and directly addressing the single-frame viewpoint limitation inherent to fixed camera arrays.

Progressive accumulation experiments on two objects demonstrated that a single 32-camera frame already achieves an F-score exceeding 0.97 at a 0.5 mm threshold, and that accumulating additional temporal frames reaches a stable convergence plateau with both objects reaching a plateau F-score of 0.984, beyond which additional frames yield diminishing returns. Across 30 independent free-fall trials with three soybean objects of approximately 8–10 mm diameter, the system achieved an overall mean error of 0.146±0.033 mm, an RMSE of 0.235±0.043 mm, and an F-score of 0.980±0.006 against an industrial structured-light scanner reference—a relative error of approximately 1.6%. Fine surface features, such as structural cracks, were resolved at a fidelity sufficient for visual defect identification. 6-DOF kinematic analysis of two objects confirmed a stable, uniformly accelerated translation with acceleration deviations of +1.6% and −0.4% relative to the standard gravitational constant, and rotation consistent with approximately constant angular velocities of 460°/s and 237°/s, demonstrating that the proposed algorithm can accurately recover both geometric shape and dynamic state from a single measurement sequence.

These results establish rigid-body multi-frame temporal integration as an efficient strategy for high-throughput, non-contact 3-D inspection of small objects in motion—a regime in which conventional industrial scanners cannot operate. Future work will focus on reducing per-object reconstruction time toward real-time operation, extending the system to low-texture and reflective surfaces through active illumination, and integrating trajectory-based sorting actuation to realize a complete inline inspection pipeline.

## Figures and Tables

**Table 1:** Hardware specifications of the PC cluster. All Intel CPUs are manufactured by Intel Corporation (Santa Clara, CA, USA); all NVIDIA GPUs by NVIDIA Corporation (Santa Clara, CA, USA).

PC	RAM	CPU	GPU
PC0	128 GB	Intel Core i9-12900KF (12th Gen)	NVIDIA GeForce RTX 3090
PC1	64 GB	Intel Core i9-14900KF	NVIDIA GeForce RTX 4070
PC2	64 GB	Intel Core i9-14900HX	NVIDIA GeForce RTX 4090 Laptop
PC3	32 GB	Intel Core i7-10857H	NVIDIA GeForce RTX 2080 Super (Max-Q)
PC4	32 GB	Intel Core i7-9700 @ 3.00 GHz	NVIDIA GeForce RTX 2080 Ti
PC5	32 GB	Intel Core i7-9700 @ 3.00 GHz	NVIDIA GeForce RTX 2080 Ti
PC6	32 GB	Intel Core i7-9700 @ 3.00 GHz	NVIDIA GeForce RTX 2080 Ti
PC7	32 GB	Intel Core i7-9700 @ 3.00 GHz	NVIDIA GeForce RTX 2080 Ti

**Table 2:** Reconstruction error at selected accumulation levels for Object 1, measured against the ATOS reference with fixed scale alignment. The Frames column indicates the number of complete 32-camera frames accumulated. The rightmost column reports the per-step SfM–MVS wall-clock time on PC0 (single run).

Images	Frames	Mean (mm)	RMSE (mm)	Max (mm)	F@0.5	Time (s)
8	—	4.790	5.369	11.428	0.004	13.2
16	—	0.137	0.191	1.299	0.731	22.3
24	—	0.521	0.561	1.600	0.486	26.3
32	1	0.115	0.199	1.610	0.978	32.3
64	2	0.172	0.230	1.620	0.983	64.7
96	3	0.124	0.201	1.617	0.984	92.9
128	4	0.111	0.195	1.614	0.984	89.1
160	5	0.108	0.192	1.607	0.984	108.1
192	6	0.132	0.207	1.618	0.983	86.9

**Table 3:** Reconstruction error at selected accumulation levels for Object 2, measured against the ATOS reference with fixed scale alignment. The rightmost column reports the per-step SfM–MVS wall-clock time on PC0 (single run).

Images	Frames	Mean (mm)	RMSE (mm)	Max (mm)	F@0.5	Time (s)
8	—	7.624	8.383	22.252	0.000	23.2
16	—	0.087	0.119	0.894	0.862	16.4
24	—	0.236	0.278	1.586	0.971	20.3
32	1	0.238	0.279	1.589	0.982	21.6
64	2	0.279	0.310	1.592	0.982	33.8
96	3	0.430	0.448	1.700	0.908	45.5
128	4	0.207	0.252	1.583	0.984	115.0
160	5	0.203	0.249	1.593	0.984	133.1
192	6	0.161	0.221	1.597	0.984	86.8

**Table 4:** Shape fidelity error statistics for 30 matched comparisons against reference 3-D models (mean ± std).

Object	n	Mean Error (mm)	RMSE (mm)	Max Error (mm)	F@0.5
Object 1	10	0.138±0.028	0.212±0.017	1.624±0.013	0.984±0.001
Object 2	10	0.132±0.037	0.203±0.019	1.599±0.016	0.985±0.000
Object 3	10	0.167±0.024	0.291±0.014	2.079±0.012	0.971±0.001
**Overall**	**30**	**0.146 ± 0.033**	**0.235 ± 0.043**	**1.768 ± 0.221**	**0.980 ± 0.006**

**Table 5:** Sub-sampling ablation on the existing Object 2 sequence (falling_009): four sub-array configurations extracted from the same 195 captured images and processed through the identical SfM–MVS pipeline, compared against the 32-camera baseline. The 8+8 dual-ring scheme could not be evaluated cleanly on this sequence due to the absence of Cam05 throughout the recording.

Configuration	Cams	Topology	RMSE (mm)	F@0.5
8-camera single equatorial ring	8	1 ring	48.62	0.000
8-camera dual-layer (upper 4 + lower 4)	8	2 layers	0.78	0.546
16-camera single equatorial ring	16	1 ring	0.91	0.209
16-camera three-layer (upper 4 + middle 8 + lower 4)	16	3 layers	0.50	0.953
**Object 2 baseline** (Table 3, single-trial best)	32	3 layers	**0.25**	**0.984**

## Data Availability

The data presented in this study are available on request from the corresponding author.
